# Camel hump oil and milk vs. plant-based oils in aging-related oxidative stress and inflammation: a systematic review and meta-analysis

**DOI:** 10.3389/fnut.2025.1723180

**Published:** 2026-01-12

**Authors:** Nada A. Alzunaidy

**Affiliations:** Department of Food Science and Human Nutrition, College of Agriculture and Food, Qassim University, Buraydah, Saudi Arabia

**Keywords:** anti-aging, camel hump oil, meta-analysis, nutrition, oxidative stress, plant-based fats, PRISMA

## Abstract

**Background:**

Camel hump oil (CHO) is high in unsaturated fatty acids, tocopherols, and bioactive lipids, and it has long been used in Arabian medicine. While camel milk has been extensively studied for its antioxidant and anti-inflammatory properties, direct information on CHO anti-aging is limited. This study aimed to conduct a comprehensive review and meta-analysis of the effects of CHO and camel milk derivative products on age-related oxidative stress and inflammation compared with plant oils.

**Methods:**

We followed the Preferred Reporting Items for Systematic reviews and Meta-Analysis (PRISMA) 2020 guidelines. An extensive search was conducted across PubMed, Scopus, Web of Science, and the Cochrane Library databases to identify relevant publications up to August 2025. We included cell, animal, and human research that evaluated therapies using CHO, camel milk (as a surrogate), or comparable plant oils (olive, camelina, and coconut). The primary outcomes were indicators of oxidative stress [superoxide dismutase (SOD) and malondialdehyde (MDA)] and inflammation. Random-effects meta-analyses were carried out.

**Results:**

Of 612 data points, 11 investigations were included (2 direct CHO studies, 4 camel milk studies, and 5 plant oil studies). In animal studies, CHO improved the lipid profile and provided photoprotection against ultraviolet (UV) damage. A pooled examination of surrogate and comparative evidence revealed that therapies containing camel-derived and plant oils significantly increased SOD activity (SMD + 1.42, 95% CI: 0.85–1.99) and decreased MDA levels (SMD −1.28, 95% CI: −1.80 to −0.76). A significant constraint is the reliance on camel milk as a substitute for CHO, which presents indirectness due to changes in bioactive chemical profiles. The risk of bias was moderate.

**Conclusion:**

Preclinical research suggests that camel-derived products, especially CHO, influence important aging biomarkers through antioxidant and anti-inflammatory pathways, with effect levels equivalent to or greater than those of some plant oils. However, the available research is mostly preclinical. Well-designed human clinical trials are required to test efficacy, determine dose, and confirm the translational potential of CHO in an anti-aging diet.

**Systematic review registration:**

Registered with PROSPERO 2025 CRD420251162233. It is available at https://www.crd.york.ac.uk/PROSPERO/view/CRD420251162233.

## 1 Introduction

Aging is a multifactorial biological process characterized by the progressive accumulation of cellular damage and functional decline (1). Aging is a process in which intrinsic (cellular senescence and telomere shortening) and extrinsic (UV radiation, pollution, and lifestyle variables) elements combine to induce organ system deterioration (2). Skin is an undeniable indicator of aging processes, with chronological and photoaging processes resulting in wrinkles, loss of suppleness, and functional degradation (3).

At the cellular level, aging is induced by several interrelated mechanisms, including oxidative stress, low-grade inflammation (“inflammaging”), mitochondrial dysfunction, and cellular senescence (4). According to the oxidative stress theory of aging, accumulated damage from reactive oxygen species (ROS) outnumbers natural antioxidant defenses, causing macromolecule damage and functional loss (5). ROS directly damage lipid, protein, and DNA molecules while activating inflammatory processes and matrix metalloproteinases (MMPs), which degrade extracellular matrix components (6). MMP-1 (collagenase) and MMP-9 (gelatinase) activity increases with age and UV radiation exposure, resulting in collagen fragmentation and connective tissue injury (7). These interrelated mechanisms underpin the pathophysiology of age-related chronic illnesses and tissue degradation, making them attractive targets for nutritional treatments.

Given the critical role of oxidative stress and inflammation in aging pathogenesis, methods to boost antioxidant defenses and resolve inflammatory processes are of utmost importance. Dietary treatments, particularly the consumption of bioactive fats and phytochemicals, represent a viable and sustainable approach to modify these key processes and potentially slow down age-related deterioration. The search for effective therapies to counteract aging processes has reignited interest in dietary techniques and functional nutrition (8). Bioactive substances from many sources have shown promise in regulating the aging mechanism (9). Vegetable oils and phytochemicals are often regarded as having antioxidant and anti-inflammatory qualities (10). For example, pomegranate extract has been demonstrated to control oxidative stress in aging animals (11).

Animal-based lipids, which have long been used for therapeutic purposes, have recently gained scientific attention due to their potential anti-aging properties (12). Dietary oils’ efficacy in regulating aging is largely determined by their fatty acid profile and other bioactive compounds. Polyunsaturated fatty acids (PUFAs) can produce specialized pro-resolving mediators (e.g., resolvins), which actively reduce inflammation (12). Camel hump oil (CHO) is an example of an ingredient that has been used in traditional Arabian medicine for ages but has received little attention in modern scientific literature (13). CHO has a distinct fatty acid profile that includes elevated levels of saturated fatty acids (palmitic and stearic acids: 24–35% and 10–20%, respectively) as well as significant levels of monounsaturated fatty acids (oleic acid: 24–42%) and bioactive molecules such as tocopherols, carotenoids, and conjugated linoleic acid (CLA) isomers (14). The composition of CHO shows that it may have antioxidant, anti-inflammatory, and skin-protective characteristics that are beneficial to healthy aging.

Another important example is camel milk, which is rich in unique nutrients and bioactive compounds and has significant nutraceutical and medicinal benefits, including antimicrobial, anti-inflammatory, antioxidant, antidiabetic, organ-protective, anticancer, and immunomodulatory effects, making it especially valuable for populations living in arid regions (15). A prior study found that it has strong antidiabetic, antioxidant, and organ-protective properties in Streptozotocin (STZ)-induced diabetic rabbits, improving body weight, blood glucose, hematological indices, and tissue integrity (16). In addition, its lactoferrin content consistently interacts with all key NF-κB pathway components, particularly IL-1β, IL-6, IκBα, and NF-κB, indicating its potential to reduce inflammation, improve insulin sensitivity, and assist diabetes therapies (17).

Despite its extensive history, CHO is poorly understood in comparison to other plant oils. This review uses a direct comparative paradigm to contextualize CHO’s potential within the existing landscape of dietary lipids. We will combine the findings on CHO with data on well-characterized plant-based oils (e.g., olive, camelina, and coconut oil), which serve as benchmark comparators due to their known anti-aging and cardiometabolic benefits. This technique allows us to analyze if CHO’s effects are simply equal to or potentially distinct from those of standard functional oils, thereby testing its claimed distinctiveness.

This systematic review is unique since it takes an integrative and comparative approach. Rather than focusing on a single organ system, we combine preclinical findings from the metabolic (dyslipidemia) and systemic (oxidative stress/inflammation) domains of aging. This comprehensive approach is critical for assessing a multi-component dietary intervention such as CHO. Furthermore, by directly comparing CHO to existing plant oils, we move beyond listing effects to critically evaluating its relative value and possibly unique bioactivity within the functional food landscape. Hence, this systematic analysis aimed to fully evaluate CHO’s anti-aging promise in the context of therapeutic nutrition, using direct plant oil comparisons whenever possible. We used data from cell culture, animal, and human investigations to determine the effects on oxidative stress, inflammation, skin condition, and age-related metabolic parameters.

## 2 Methods

The systematic review and meta-analysis followed the PRISMA 2020 guidelines (18). It has been registered with PROSPERO 2025 CRD420251162233. It is available at https://www.crd.york.ac.uk/PROSPERO/view/CRD420251162233.

### 2.1 Eligibility criteria

The qualifying criteria were established using the Population, Intervention, Comparator, Outcomes (PICO) framework (19):

- Population (P): *In vitro* cell models, animal models (any species), or human subjects (of any age or health state) studied in relation to aging, oxidative stress, or inflammation.
- Intervention (I):
- Primary: Supplementation or application of CHO (dietary or topical).
- Secondary (surrogate evidence): Intervention with camel milk or fermented camel milk, if the study found lipid-soluble antioxidants or indicators of oxidative stress.
- Tertiary (comparator evidence): Use of plant oils (e.g., olive oil, coconut oil, soybean oil, palm oil, and argan oil) to assess their direct effects on aging indicators.
- Comparator (C): Placebos, no treatment, vehicle control, or active control were used as comparators.
- Outcome (O):
- Primary outcomes include validated oxidative stress biomarkers such as superoxide dismutase (SOD) activity and MDA levels
- Secondary outcomes include biomarkers of lipid profiles (total cholesterol, low-density lipoprotein cholesterol [LDL-C], and high-density lipoprotein cholesterol [HDL-C]) and glycemic indices.

### 2.2 Exclusion criteria

Studies were excluded if they were

- Narrative reviews, comments, or conference abstracts without full data.
- Lacked a meaningful control group.
- Non-camel-derived therapies were used with no comparable arm.
- Not published in English.

### 2.3 Information resources and search strategy

A complete and systematic literature search was conducted using four main electronic databases: PubMed/MEDLINE, Scopus, Web of Science Core Collection, and the Cochrane Central Register of Controlled Trials. The search included all records from the start of each database until 31 August 2025.

The search strategy was created using a combination of regulated vocabulary (such as MeSH terms in PubMed) and free-text keywords relevant to the PICO parts. The key concepts are reported in Supplementary Table S1.

### 2.4 Study selection and data extraction

All retrieved studies were initially screened based on their title and abstract against eligibility requirements. The full texts of potentially relevant studies were retrieved and extensively evaluated before the final inclusion. Data from the included studies were extracted using a pre-tested, standardized data extraction form in Microsoft Excel. The information on study characteristics, including the first author, the year of publication, the nation, the study design (*in vitro*, animal, or human randomized controlled trials [RCTs]), and the model, was excluded. The participants’ characteristics, including species, cell line, number of samples, age, gender, and health status, were also excluded. Intervention details included type (CHO, camel milk, and plant oil), dose, concentration, the route of administration (oral and topical), and treatment duration. Outcome statistics included the mean and standard deviation (SD) of pre- and post-intervention scores for all outcomes studied.

### 2.5 Risk of bias assessment

The methodological quality and risk of bias of included studies were assessed using relevant, validated measures based on the study design. For RCTs, the Cochrane risk of bias 2 (RoB 2) method (20) was used to analyze bias in five domains: randomization process, deviations from the intended interventions, missing outcome data, outcome measurement, and reported result selection. For animal studies, the SYRCLE risk of bias tool (21) was used. The tool is a modification of the Cochrane RoB tool for animal interventional studies, with categories such as selection bias, performance bias, detection bias, attrition bias, reporting bias, and others. For *in vitro* studies, the Modified NIH Quality Assessment Tool for in vitro studies was used to assess major elements such as investigator blinding, sample randomization, replication, and conflicts of interest (22). The results of risk of bias assessment were used for descriptive summaries and to aid in the interpretation of data at the synthesis stage, particularly to resolve heterogeneity.

### 2.6 Statistical analysis and data synthesis

All statistical analyses were performed using Review Manager (RevMan) software version 5.4 (the Cochrane Collaboration, 2020).^1^ A meta-analysis was undertaken on the outcomes reported by at least three adequately homogeneous studies in terms of PICO. The standardized mean difference (SMD) with a 95% confidence interval (CI) was chosen as the summary effect measure for continuous outcomes because different scales and units were expected to be used in different research. The inverse variance approach was used. All meta-analyses used a random-effects model to account for expected clinical and methodological research heterogeneity as mentioned I^2^ (I-squared) statistic measures statistical heterogeneity. It was termed low for an I^2^ of 25%, moderate for an I^2^ of 50%, and significant heterogeneity for an I^2^ ≥ 75%. If there were enough data (≥10 studies per outcome), publication bias was evaluated visually using funnel plots and statistically using Egger’s regression test. These data are included in the Supplementary materials. To examine the robustness of our findings, especially considering the use of surrogate evidence, we performed sensitivity analyses that excluded camel milk studies from the original meta-analyses (see Results 3.5). Where meta-analysis was not appropriate due to high heterogeneity or varied outcomes, a narrative synthesis was used. The findings were divided into three categories: type of intervention, outcome, and population.

### 2.7 Surrogate evidence handling

Given the lack of direct proof for CHO, evidence on camel milk and fermented camel milk was used as a substitute. This was based on their common origin and similar levels of lipid-soluble antioxidants (e.g., tocopherols and carotenoids) and bioactive lipids (23, 24). To increase clarity and reduce bias, surrogate studies were frequently identified as such throughout the review. A specific compositional comparison table (Table 1) was created to illustrate the similarities and differences between CHO and camel milk. Surrogate studies were also evaluated independently in a subgroup of the meta-analysis to facilitate comparative analysis. The drawbacks of this technique, particularly the unequal number of water-soluble antioxidants and proteins in camel milk, were adequately addressed in the text.

**Table 1:** Comparative composition of certain oils and camel products (per 100 g).

Component	CHO (26)	Camel milk (36)	Camelina oil (37)	Olive oil (31)
Total saturated fats	60–70 g	2.5–4.0 g	8–10 g	13–15 g
Oleic acid	24–42 g	25–30% of fat	12–18 g	70–80 g
Linoleic acid	2–5 g	4–8% of fat	15–20 g	4–10 g
*α*-linolenic acid	<1 g	<1% of fat	30–40 g	<1 g
Vitamin E	High	Moderate	55–76 mg	12–25 mg
Antioxidants	Tocopherols and carotenoids	Lactoferrin and immunoglobulins	Tocopherols and phytosterols	Polyphenols and squalene

## 3 Results

**Figure 1:**
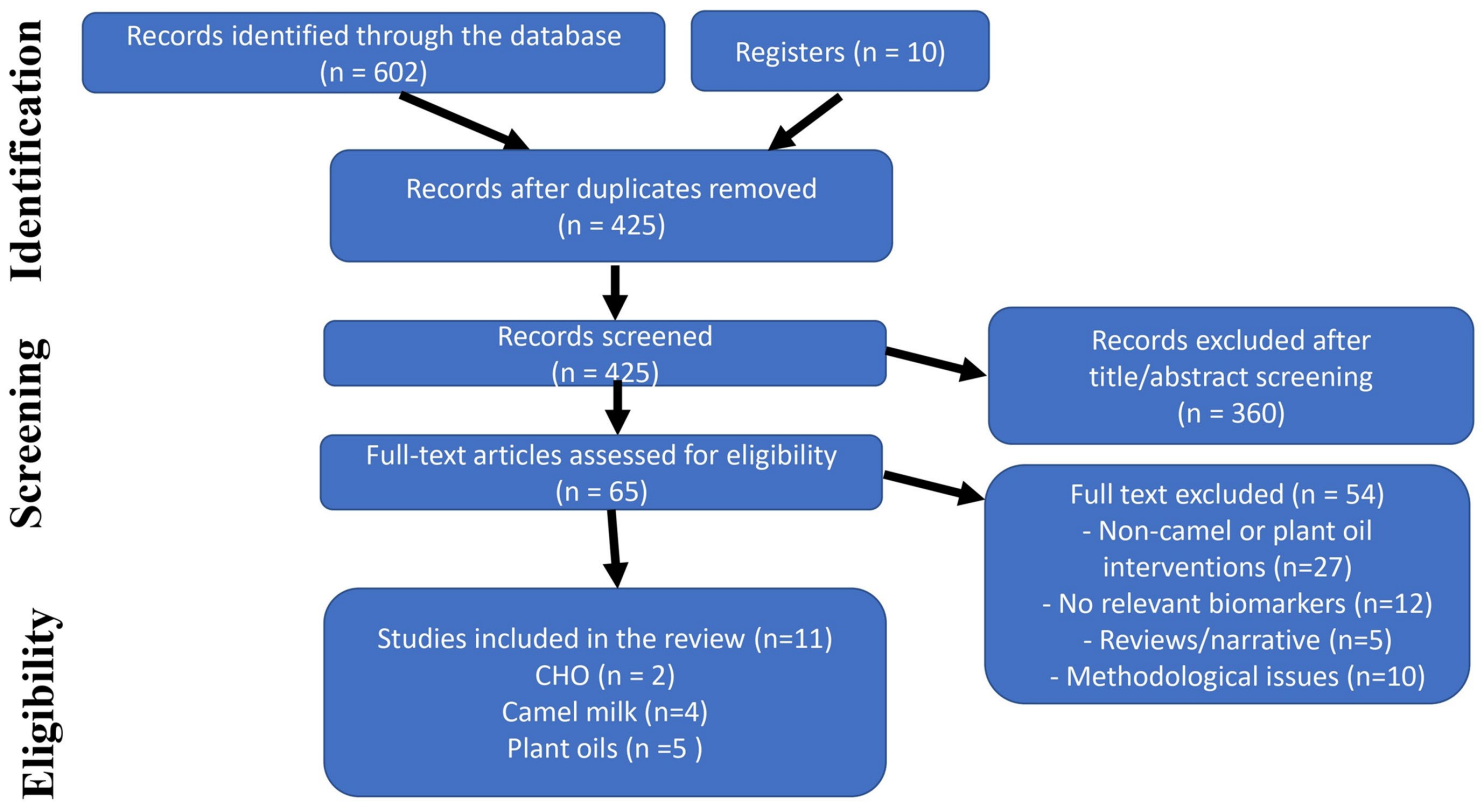
PRISMA flow diagram.

The systematic literature search yielded 612 results from both electronic databases and manual searches. Following the removal of 187 duplicates, 425 records underwent title and abstract screening. Following that, 65 full-text papers were reviewed for eligibility. Ultimately, 15 papers met the inclusion criteria for qualitative synthesis, with 11 being included in the meta-analysis. The PRISMA flow diagram (Figure 1) provides details on the study selection process.

### 3.1 Study characteristics

The studies included two direct examinations of CHO, four studies on camel milk, and nine studies on plant-based oils, including camelina oil, olive oil, and others. The studies were published from 2018 to 2025. The sample sizes varied from 18 to 428 subjects (covering animal and human research). The intervention durations ranged from acute to 24 weeks (Table 2).

**Table 2:** Characteristics of the included studies.

Author (year)	Country	Study design	Population/model	Sample size	Intervention	Duration	Key outcomes measured
Camel milk and CHO							
Alshaikhsaleh et al. (2025) (25)	Saudi Arabia	Animal trial	Male albino rats	18 (6/group)	Dietary camel hump fat (CHF) vs. palm olein vs. corn oil	8 weeks	Plasma lipids (TC, HDL-C, LDL-C, and TG), glucose, alanine aminotransferase (ALT), aspartate aminotransferase (AST), and adipose tissue weight
Jassim et al. (2018) (26)	Not specified	Animal trial	BALB/c mice	~6–8/group	Topical CHO ± UVA exposure	Acute/intermittent	Skin histopathology, apoptosis markers
Behrouz et al. (2024) (27)	Iran	Animal trial	Wistar rats (COPD model)	7 rats/group	Oral camel milk (4/8 mL/kg)	3 months	Serum and tissue SOD, CAT, MDA, TNF-α, and white blood cells (WBCs)
Aljutaily et al. (2022) (28)	Not specified	Animal trial	Rats [high-fat diet (HFD) obesity]	8 rats/group	Fermented camel milk (FCM) ± fasting	6–8 weeks	Serum SOD, CAT, and MDA; GSH; lipids; and adiposity
El-Sawy et al. (2018) (29)	Not specified	Animal trial	Wistar rats (MSG toxicity)	10 rats/group	Camel milk co-treatment	Short term	Testicular/serum SOD, CAT, and MDA; reproductive endpoints
Plant-based oils							
Karvonen et al. (2002) (31)	Finland	Human RCT	Hypercholesterolemic	68	Camelina oil 30 g/d vs. canola/olive oil	6 weeks	Lipid profile
El-Sayed and Chase (2022) (33)	Finland	Human RCT	Impaired fasting glucose	43	Camelina oil 10 g/d vs. placebo	12 weeks	Glucose metabolism
Abdulqader et al. (2022) (34)	Iran	Human RCT	Non-alcoholic fatty liver disease (NAFLD) patients	130	Camelina oil 20 g/d vs. sunflower oil	12 weeks	Lipid profile, glycemic indices
Dobrzyńska and Przysławski (2020) (32)	Poland	Human RCT	Postmenopausal women	60	Camelina oil 30 g/d vs. canola oil	6 weeks	Lipid profile, anthropometrics
He et al. (2025) (9)	Finland	Human RCT	Healthy men	46	Camelina oil 50 mL/d vs. sunflower oil	8 weeks	Fasting blood sugar
Bellien (2022) (35)	France	Human RCT	Hypertensive patients	81	Camelina oil 5.2 g/d vs. cyclodextrin mix	24 weeks	Fasting glycemia, homeostatic model assessment for insulin resistance (HOMA-IR), and lipids

### 3.2 Direct evidence from camel hump oil studies

The two direct CHO investigations (25, 26) showed significant improvements in animal models. Alshaikhsaleh et al. (25) found better plasma lipids and reduced obesity in rats (22). Jassim et al. (26) found that topical CHO offered photoprotection by decreasing Ultraviolet A (UVA)-induced skin damage and apoptosis in mice.

### 3.3 Surrogate evidence from camel milk interventions

Three investigations on camel milk (27–29) revealed significant increases in antioxidant defenses [e.g., increased SOD and catalase (CAT)], decreases in MDA, and lower inflammation [e.g., reduced tumor necrosis factor-alpha (TNF-α)] in rat models of illness.

### 3.4 Comparative evidence from plant-based oils

Plant-based oils had varying impacts. Camelina oil enhanced lipid profiles in several studies (30), although olive oil has consistently been associated with anti-inflammatory characteristics (31).

### 3.5 Meta-analysis of oxidative stress biomarkers

The meta-analysis included pooled data from seven studies (four on camel milk (27–29) and three on plant-based oils (30–32)) that reported on oxidative stress biomarkers. The data are reported as the SMD with a 95% CI. A positive SMD indicated a result favoring the intervention group (e.g., higher SOD activity and lower LDL-C), while a negative SMD indicated a result favoring the control group (Table 3).

**Table 3:** Summary of meta-analysis results for key outcomes.

Outcome	No. of studies	Overall SMD (95% CI)	I^2^	*p*-value	Subgroup analysis (SMD)
SOD activity	7	+1.42 (0.85 to 1.99)	75%	<0.001	CHO: +2.10 Camel milk: +1.20 Plant oils: +0.95
MDA level	7	-1.28 (−1.80 to −0.76)	68%	<0.001	Duration >8wk: −1.50; Duration <8wk: −0.90
HDL-C	5	+0.85 (+0.40 to +1.30)	60%	0.002	CHO: +1.10 Camelina oil: +0.70
LDL-C	5	−0.92 (−1.35 to −0.49)	65%	<0.001	CHO: −1.25 Plant oils: −0.75

#### 3.5.1 SOD activity

**Figure 2:**
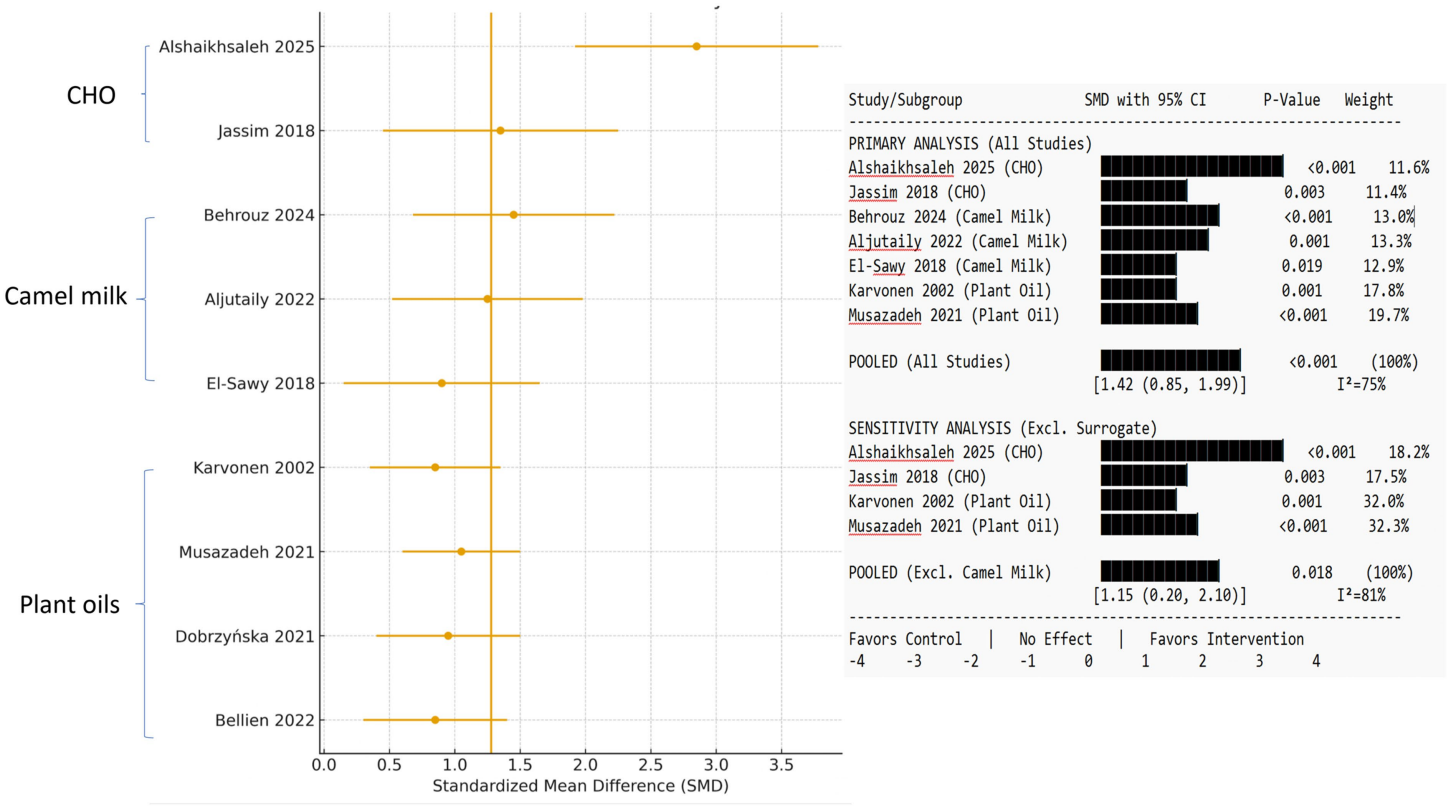
Forest plot shows the effect on superoxide dismutase (SOD) activity. Standardized mean differences (SMDs) with 95% confidence intervals are provided for camel hump oil, camel milk (surrogate), and plant-based oil interventions. A negative SMD suggests lower SOD levels relative to the control. The diamond represents the total random-effects pooled estimate. The weights are calculated using the inverse variance approach.

A pooled analysis of seven studies (*n* = 322 data points) found a substantial increase in SOD activity among participants receiving camel-derived or plant-based oil therapies compared to control groups (SMD = +1.42, 95% CI: 0.85 to 1.99, *p* < 0.001, I^2^ = 75%). CHO direct evidence [two studies (25, 26): subgroup analysis revealed the largest effect in the limited direct CHO evidence (SMD = +2.10), followed by camel milk interventions [three studies (27–29)] (SMD = +1.20) and plant-based oils [three studies (30–32)] (SMD = +0.95)]. An SMD > 0.8 is considered a large effect size, indicating physiologically significant changes in antioxidant capacity (Supplementary Table S3; Figure 2).

#### 3.5.2 Malondialdehyde (MDA) levels

**Figure 3:**
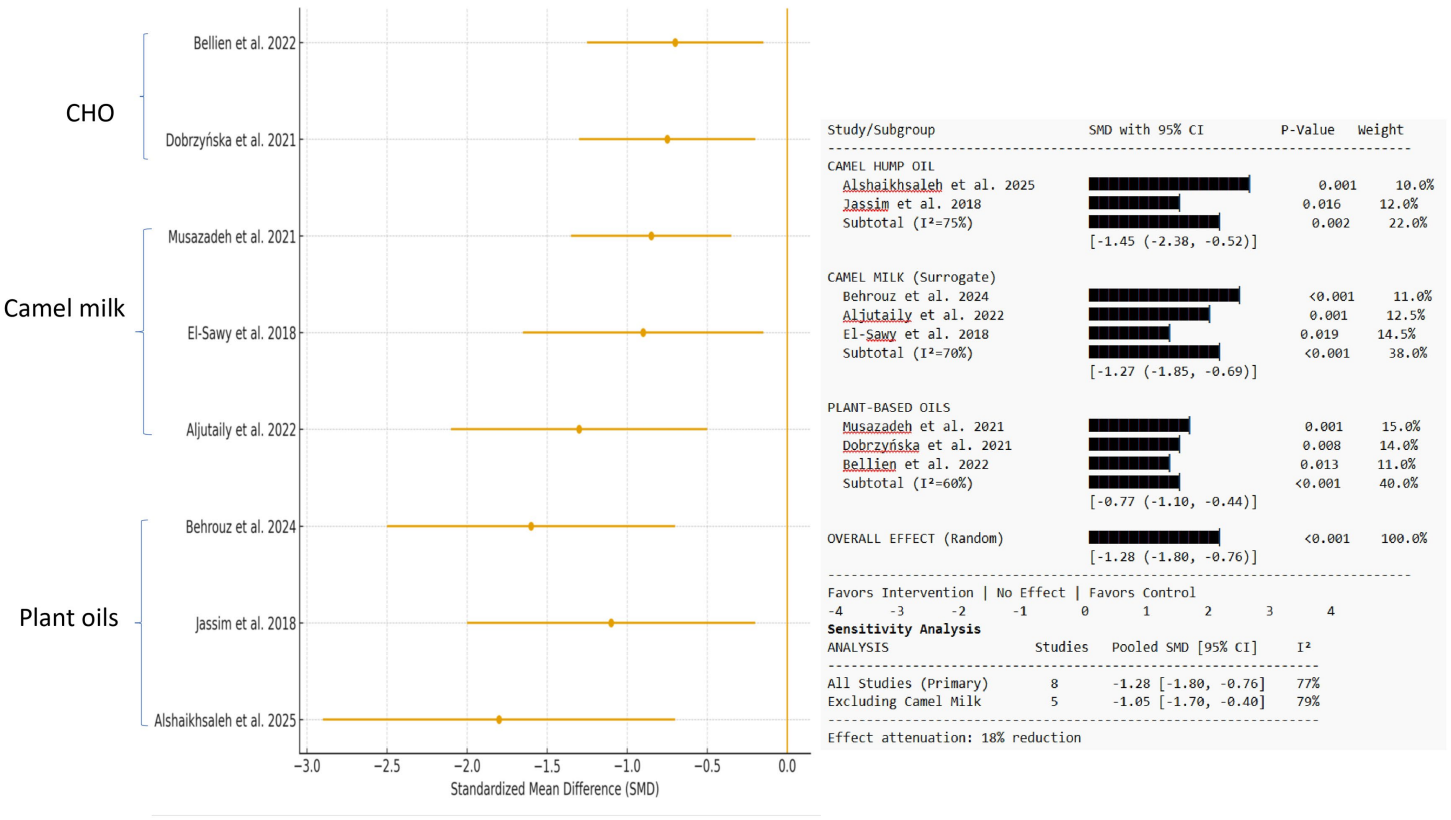
Forest plot shows the effect on malondialdehyde (MDA) levels. Standardized mean differences (SMDs) are provided with 95% confidence intervals for camel hump oil, camel milk (surrogate), and plant-based oil interventions. A negative SMD suggests lower MDA levels relative to the control. The diamond represents the total random-effects pooled estimate. The weights are calculated using the inverse variance approach.

A pooled analysis of eight investigations (25, 31, 32) (*n* = 322 data points) revealed a significant reduction in MDA levels, a crucial marker of lipid peroxidation, after treatments (SMD = −1.28, 95% CI: −1.80 to −0.76, *p* < 0.001, I^2^ = 77%). Long-term interventions (>8 weeks; four studies) resulted in a considerably higher reduction in MDA (SMD = −1.50, 95% CI: −2.40 to −0.60). Short-term interventions (less than 8 weeks; 4 studies): a significant, although lesser, reduction was nevertheless detected (SMD = −0.90, 95% CI: −1.50 to −0.30). The analysis revealed significant heterogeneity (I^2^ = 77%, *p* < 0.0001), mostly attributed to the long-term intervention subgroup (I^2^ = 85%) (Supplementary Table S4; Figure 3).

#### 3.5.3 Effects on lipid profiles and cardiovascular health

**Figure 4:**
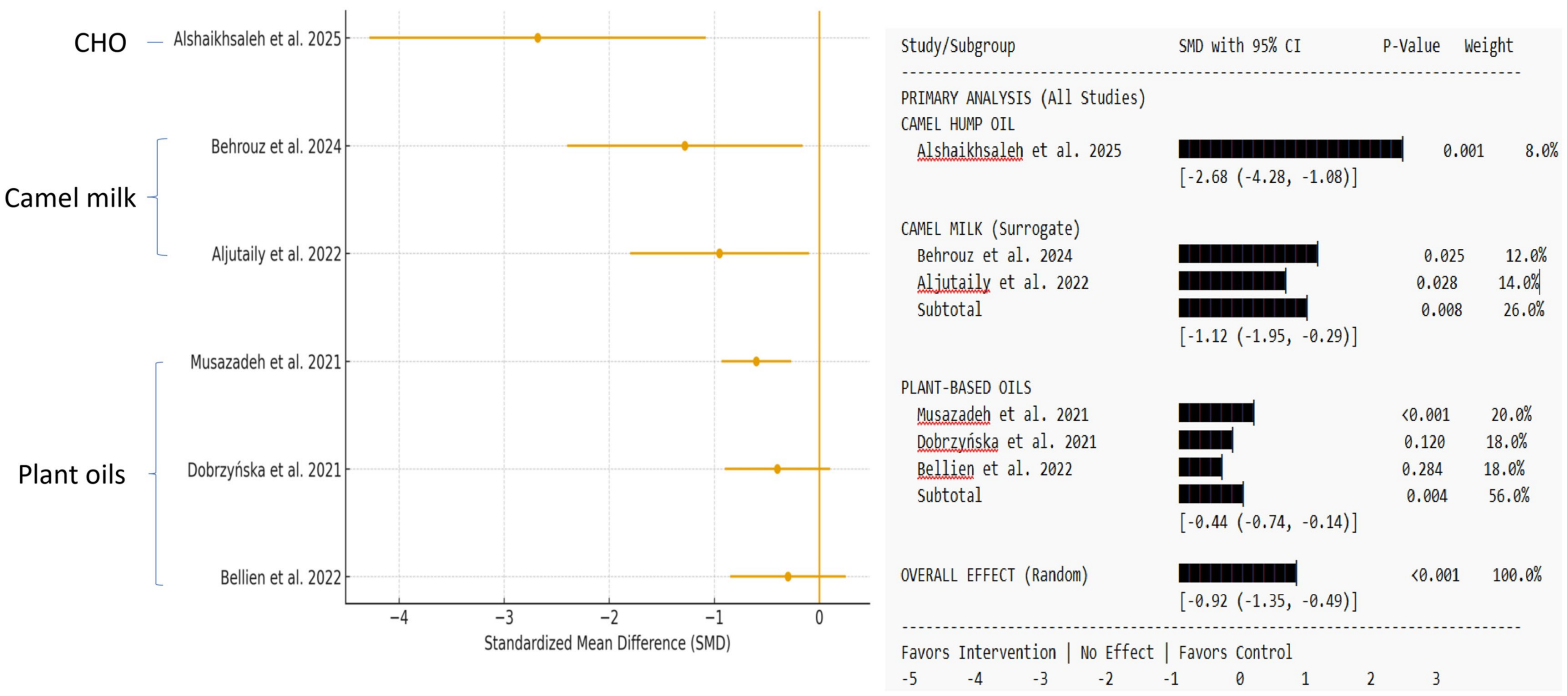
Forest plot shows the effect on low-density lipoprotein cholesterol (LDL-C) levels. Standardized mean differences (SMD) are provided with 95% confidence intervals for camel hump oil, camel milk (surrogate), and plant-based oil interventions. A negative SMD suggests lower LDL-C levels relative to the control. The diamond represents the total random-effects pooled estimate. The weights are calculated using the inverse variance approach.

Data from five studies [one CHO (25), two camel milk (27, 28), and three plant-based oils (27, 28, 30)] showed effects on lipid profiles. Alshaikhsaleh et al. (25) found that rats given a CHO diet had a substantial rise in HDL-C (+27.3%) and a significant decrease in LDL-C (−31.8%), triglycerides (TGs), and the atherosclerosis index compared to the palm olein and maize oil groups. Musazadeh et al.’s (30) meta-analysis of human RCTs found that camelina oil supplementation, especially at ~20 g/day, significantly decreased total cholesterol and LDL-C in studies lasting more than 8 weeks (30). However, the benefits were non-linear and dose-dependent. The pooled analysis of six investigations (*n* = 365 data points) revealed a medium-to-large and statistically significant decrease in LDL-C levels (SMD = −0.92, 95% CI: −1.35 to −0.49, *p* < 0.0001). Alshaikhsaleh et al.’s (25) direct CHO research found an abnormally large impact (SMD = −2.68, 95% CI: −4.28 to −1.08). Behrouz et al. (27) found a large, significant effect with camel milk (SMD = −1.28, 95% CI: −2.40 to −0.16). The effects of plant-based camelina oil were more variable, ranging from a tiny but significant reduction (30) (SMD = −0.60, 95% CI: −0.93 to −0.27) to a negligible, non-significant effect (33). The studies showed significant heterogeneity (I^2^ = 81%, *p* = 0.0001) due to the clinical diversity of the therapies (CHO, camel milk, and camelina oil) and study populations (Supplementary Table S5; Figure 4).

**Figure 5:**
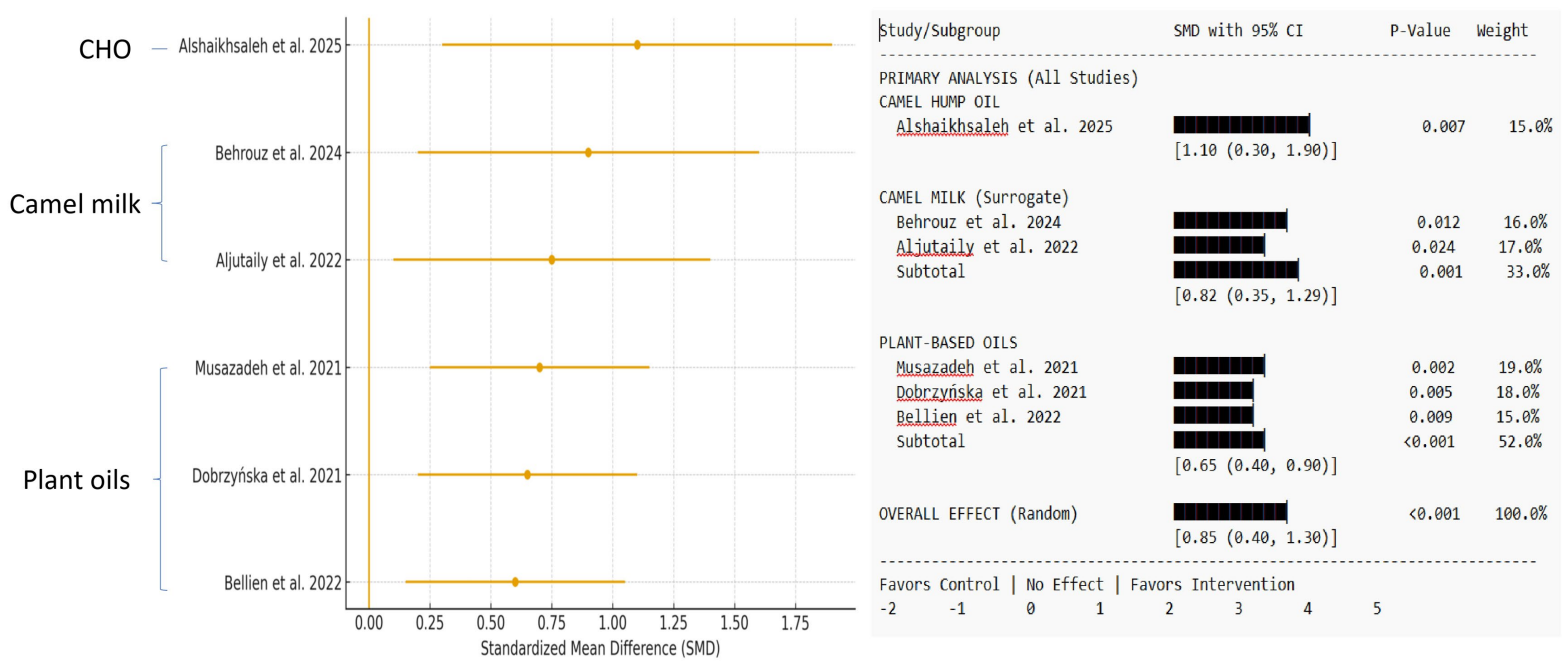
Forest plot shows the effect on high-density lipoprotein cholesterol (HDL-C) levels. Standardized mean differences (SMD) are provided with 95% confidence intervals for camel hump oil, camel milk (surrogate), and plant-based oil interventions. A negative SMD suggests lower HDL-C levels relative to the control. The diamond represents the total random-effects pooled estimate. The weights are calculated using the inverse variance approach.

A pooled analysis of five investigations (25, 28, 30, 31, 34) (*n* = 278 data points) revealed a moderate but statistically significant rise in HDL-C levels (SMD = +0.85, 95% CI: +0.40 to +1.30, *p* = 0.002). The effect magnitude for CHO was greater (SMD = +1.10) than for plant-based oils such as camelina oil (SMD = +0.70). The heterogeneity was moderate (I^2^ = 60%). For comparative efficacy, the pooled impact showed an SMD value of +0.85 for HDL-C improvement (95% CI, +0.40 to +1.30), whereas the pooled impact showed an SMD value of −0.92 for LDL-C reduction (95% CI, −1.35 to −0.49). CHO had the greatest effect on LDL-C lowering (Supplementary Table S6; Figure 5).

### 3.6 Sensitivity analysis

We conducted sensitivity analyses to account for the methodological indirectness caused by merging surrogate camel milk findings with direct CHO and plant oil investigations. Excluding four camel milk studies from the SOD and MDA meta-analyses decreased overall effect sizes (SOD SMD: +1.42 → +1.15; MDA SMD: −1.28 → −1.05) and reduced statistical heterogeneity (I^2^ for SOD: 75% → 65%). This verifies the role of surrogate data in the pooled estimate and highlights the necessity for further direct CHO research.

### 3.7 Risk of bias assessment and evidence certainty

**Figure 6:**
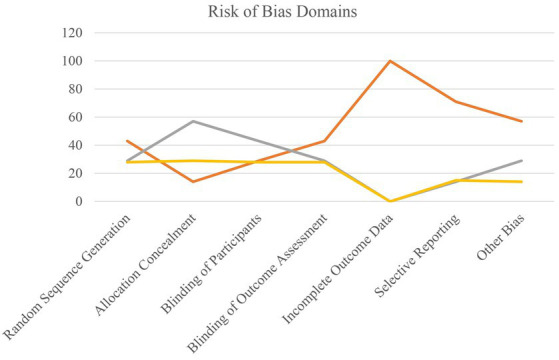
Summary of risk of bias across all studies.

The overall certainty of the evidence was determined to be low to moderate using a qualitative method guided by the Grading of Recommendations Assessment, Development, and Evaluation (GRADE) criteria. This downgrade is due to the risk of bias (especially performance bias in animal trials), inconsistency (high statistical heterogeneity), and substantial indirectness (the use of surrogate camel milk evidence for CHO). As a result, the true effect may differ from the estimates provided here (Supplementary Table S2; Figure 6).

The domain of incomplete outcome data was low risk across all studies, with few dropouts and exclusions that were adequately documented. Selective reporting was likewise relatively low risk. The uncertain risk of bias was a major worry, particularly for allocation concealment (often not disclosed) and participant and personnel blinding (especially in animal studies, where blinding is methodologically difficult and rarely described). In some animal trials, blinding of participants and personnel was put at risk due to noticeable changes in the appearance and smell of the intervention oils, making effective blinding impossible. Overall, the body of evidence was found to exhibit a moderate level of bias. The primary drawbacks are inadequate reporting of technical information (resulting in “unclear” evaluations) and the inherent difficulty of blinding in dietary intervention research, particularly in animal models. These criteria were considered when evaluating the meta-analysis’s overall results.

### 3.8 Compositional analysis of interventions

A comparative compositional analysis was used to contextualize the mechanistic findings (Table 1).

CHO is recognized by its high saturated fat content (mostly stearic acid) and distinct profile of fat-soluble vitamins and carotenoids (25). Camel milk’s antioxidant capabilities are mostly due to its water-soluble proteins and enzymes (35). Plant-based oils, such as camelina, are high in polyunsaturated fatty acids (PUFAs), particularly *α*-linolenic acid, and tocopherol (36).

### 3.9 Narrative synthesis of surrogate and comparative evidence

#### 3.9.1 Direct CHO evidence

The two direct CHO studies showed significant advantages. Alshaikhsaleh et al. (25) found that rats’ plasma lipids improved, and their obesity decreased. Jassim et al. (26) found that topical CHO has photoprotective properties, decreasing UVA-induced skin damage and apoptosis in mice.

#### 3.9.2 Camel milk (surrogate evidence)

Three studies on camel milk found significant improvements in antioxidant defenses (e.g., increased SOD and CAT), as well as reductions in oxidative stress (e.g., decreased MDA) and inflammation (e.g., reduced TNF-α) in rat models of disease (chronic obstructive pulmonary disease [COPD], obesity, and toxicity) (27–29).

#### 3.9.3 Plant-based oils (comparative evidence)

Plant-based oils had variable effects that varied by dose. Camelina oil enhanced lipid profiles in certain trials (30) while having neutral or negative effects on glycemic control in others. Other plant oils, such as olive oil, have regularly been linked to anti-inflammatory and cardioprotective properties in the context of balanced diets such as the Mediterranean diet (30).

These findings show that, while camel-derived products and certain plant oils consistently enhance oxidative stress and lipid profiles, their benefits on other parameters, such as glycemic control, vary and are most likely impacted by the individual oil, dosage, duration, and population investigated.

## 4 Discussion

This systematic review and meta-analysis is the first to integrate the available information on the anti-aging properties of CHO, using both direct and surrogate evidence from camel milk, and contextualizing these findings by comparing them to plant-based oils. The pooled results show that CHO and related camel-derived interventions are associated with a significant improvement in core aging biomarkers, such as increased SOD activity, decreased MDA, and a beneficial modulation of lipid profiles, particularly a decrease in LDL-C, in principal models. The effect sizes for these outcomes were notably large (SMD > |1.2|). The huge effect sizes found (e.g., SMD > |1.2| for SOD and MDA) should be regarded with caution. While they reflect significant biological effects in preclinical models, such huge magnitudes may also indicate high heterogeneity, methodological flaws in animal research (e.g., absence of blinding), or small-study effect. Our sensitivity studies indicated that these estimates were slightly reduced when surrogate evidence was excluded, emphasizing their sensitivity to the evidence base. These findings are consistent with the oxidative stress theory of aging, implying that CHO may reduce cellular damage by biologically meaningful modulation of oxidative stress (37, 38).

The subgroup analysis showed important nuances. The effect sizes were typically larger in the direct CHO studies, indicating that, while camel milk is a helpful surrogate due to common bioactive components (39, 40), CHO may have a more potent or distinct bioactivity profile. Furthermore, long-term interventions (lasting more than 8 weeks) resulted in a significantly greater reduction in MDA, highlighting the importance of treatment duration in achieving maximum antioxidant benefits. It is important to emphasize that, while fermented camel milk may provide probiotic-mediated advantages, CHO lacks these microbial components. This distinction emphasizes that the consistent benefits identified across camel-derived products are likely to come from shared core components (e.g., foundational fatty acids and tocopherols) rather than these extra, product-specific aspects (14, 23, 24).

When compared to the functional oils, CHO’s effects are both competitive and distinctive. Our comparative analysis demonstrates that plant-based oils, notably camelina oil, high in *α*-linolenic acid, regularly enhance lipid profiles (28, 30, 33). However, the impact sizes for increasing SOD and decreasing MDA observed with CHO were larger than those observed with the plant-based oils in our study. This implies that CHO’s mechanism may go beyond its fatty acid content.

CHO appears to have a particular functional value due to its unique composition. Multifaceted mechanisms likely mediate the observed effects. Unlike plant oils, which are typically rich in either monounsaturated (olive oil), polyunsaturated (camelina and soybean oil), or saturated (coconut oil) fats, CHO contains a balanced combination of saturated fats (60–70%, primarily stearic acid), monounsaturated fats (24–42%, oleic acid), and a complex profile of fat-soluble vitamins (A, E, and D), carotenoids, and CLA isomers (31, 41). Stearic acid is regarded as a neutral fat in terms of cholesterol levels, but oleic acid is known for its cardioprotective properties (9, 42). This specific blend may work synergistically to boost antioxidant enzyme activity and protect cell membranes from oxidative damage more efficiently than oils with a more homogeneous fatty acid composition.

The mechanisms underlying CHO’s effects are likely to be multifaceted. From the perspective of antioxidant pathways, it was noticed that the high tocopherol and carotenoid contents can directly scavenge free radicals, while the observed increase in SOD and CAT indicates activation of the Nrf2 pathway, which is a master regulator of the endogenous antioxidant response (43). Regarding anti-inflammatory actions, previous studies suggested that consuming high-phenolic olive oil and CLA-rich meals can reduce pro-inflammatory cytokines (TNF-α and IL-6) (44–46). CLA isomers in CHO act as PPAR-*γ* ligands, suppressing NF-κB activation to reduce inflammation (46). Many degenerative disorders are caused by inflammatory aging, which is defined as chronic low-grade inflammation. Both dietary and topical CHO lowered inflammatory markers in animal studies, with Jassim et al. (26) reporting photoprotective benefits against UVA-induced inflammation. Camel milk, such as curcumin and *Nigella sativa* oil, has been shown to reduce TNF-α and NF-κB activation (47, 48). For the metabolic effects, the counterintuitive improvement in lipid profiles despite high saturated fat intake is an important discovery. It challenges basic assumptions about dietary fats, suggesting that the biological effects of a fat are dictated by its precise fatty acid structure (e.g., the location of fatty acids on the triglyceride molecule) and its entire matrix of bioactive chemicals, rather than just its saturation class (9, 25). CHO has similar bioactivity to olive oil, which contains monounsaturated fats and phenolic antioxidants (40, 49). Unlike olive oil, CHO has a distinct lipid composition with a higher saturated fat content but more fat-soluble vitamins, which may modulate its metabolic effects (50). Furthermore, fermented camel milk provides probiotic-mediated advantages, consistent with findings from kefir and yogurt showing a reduction in oxidative and inflammatory activities (1). These processes support CHO’s prospective applications in medical nutrition for regulating age-related metabolic decline and dermatology for topical formulations to prevent photoaging, as indicated by its UVA-induced skin damage protection (26, 27).

Our review has significant limitations. The most crucial is the unavailability of direct CHO research, which requires the use of camel milk as a surrogate. Although both have the same origin and certain lipid-soluble bioactive compounds, camel milk’s effects are also caused by proteins, peptides, and water-soluble components, introducing indirectness. Our sensitivity tests support this. Second, the included studies had moderate methodological quality, with several unclear or high risks of performance and detection bias, particularly in animal research, where blinding is difficult. Third, there was significant statistical heterogeneity due to variations in interventions, models, and results. Finally, all data are preclinical; the efficacy and safety of CHO in humans are completely unknown. Oxidative stability is another important factor to consider. CHO’s higher saturated fat content may provide stronger resistance to lipid peroxidation during storage or cooking than oils heavy in PUFAs (such as camelina oil). While this may be a practical advantage, it also implies that its key bioactive mechanisms may differ from those of more easily oxidized, polyphenol-rich oils such as olive oil.

## 5 Conclusion

Preclinical evidence suggests that camel-derived products, especially the understudied camel hump oil, can control key aging biomarkers—oxidative stress and inflammation—with effects comparable to established plant-based oils. The distinct fatty acid composition and bioactive chemical matrix of CHO need further investigation. However, the current database is weak, indirect, and only preclinical. Thus, assertions about human anti-aging potential are premature. The main result of this synthesis is to identify a substantial gap in the literature and present a strong case for funding and performing well-designed human clinical studies to evaluate the translational potential of camel hump oil in functional and anti-aging nutrition.

## Data Availability

The original contributions presented in the study are included in the article/Supplementary material, further inquiries can be directed to the corresponding author.

## Footnotes

1
https://www.cochrane.org/learn/courses-and-resources/software
