# Correction: Camel hump oil and milk vs. plant-based oils in aging-related oxidative stress and inflammation: a systematic review and meta-analysis

**DOI:** 10.3389/fnut.2026.1788975

**Published:** 2026-02-02

**Authors:** Nada A. Alzunaidy

**Affiliations:** Department of Food Science and Human Nutrition, College of Agriculture and Food, Qassim University, Buraydah, Saudi Arabia

**Keywords:** anti-aging, camel hump oil, meta-analysis, nutrition, oxidative stress, plant-based fats, PRISMA

An incorrect number was provided for Qassim University, represented by the Deanship of Graduate Studies and Scientific Research. The incorrect number was (2025-W-1-BSRC-54521). The correct number is (2024-W-1-BSRC-54521) during the academic year 1447AH/2026AD.

The **Funding** statement was incorrectly labelled. No funding was received for this work/and or its publication. The correct **Funding** Statement appears below.

## Funding

The author(s) declared that financial support was not received for this work and/or its publication.

An **Acknowledgments** statement was incorrectly labelled as the **Funding** statement. The correct **Acknowledgments** statement appears below.

## Acknowledgments

The author gratefully acknowledges Qassim University, represented by the Deanship of Graduate Studies and Scientific Research, on the financial support for this research under the number (2024-W-1-BSRC-54521) during the academic year 1447AH/2026AD.

The original version of this article has been updated.

