# Supplementary material for: Camel hump oil and milk vs. plant-based oils in aging-related oxidative stress and inflammation: a systematic review and meta-analysis
Source: Front Nutr. 2026 Jan 12;12:1723180. doi: 10.3389/fnut.2025.1723180 (PMC12832261; doi:10.3389/fnut.2025.1723180)
Supplement: Supplementary file 1 [file Table_1.docx]

**Supplementary Table 1:** The key concepts for the search strategy.

| Concept | Type | Keywords |
| --- | --- | --- |
| 1 | CHO and Products | “CHO" OR "camel fat" OR "camel adipose tissue”. |
| 2 | Camel Milk | “Camel milk” OR “fermented camel milk" OR "camel dairy”. |
| 3 | Ageing and Oxidative Stress | "anti-ageing" OR "aging" OR "oxidative stress" OR "reactive oxygen species" OR "inflammation". |
| 4 | Plant-Based Comparators | "olive oil," "coconut oil," "soybean oil," "argan oil," or "plant oil". |
| Boolean operators (AND, OR) were used to integrate these ideas. The search strategy was customized to match the syntax of each database. | | |

**Supplementary Table 2:** Traffic Light Plot of Risk of Bias Assessment for Individual Studies.

| Risk of Bias Domain | Alshaikhsaleh 2025 (25) | Jassim 2018 (26) | Behrouz 2024 (27) | Aljutaily 2022 (28) | El-Sawy 2018 (29) | Karvonen 2002 (31) | Musazadeh 2021 (30) |
| --- | --- | --- | --- | --- | --- | --- | --- |
| Random Sequence Generation | Unclear | Unclear | Low | Low | Unclear | Low | Low |
| Allocation Concealment | Unclear | N/A | Unclear | Unclear | Unclear | Unclear | Unclear |
| Blinding of Participants /Personnel | High | High | Unclear | Unclear | Unclear | Low | Low |
| Blinding of Outcome Assessment | Unclear | Unclear | Low | Unclear | Unclear | Low | Low |
| Incomplete Outcome Data | Low | Low | Low | Low | Low | Low | Low |
| Selective Reporting | Low | Low | Low | Low | Low | Unclear | Low |
| Other Sources of Bias | Unclear | Unclear | Low | Low | Unclear | Low | Low |

*SYRCLE tool used for animal studies; Cochrane RoB 2.0 for human RCTs.*

**Supplementary Table 3:** Forest Plot Data of SOD Activity.

| Study / Subgroup | SMD | 95% CI Lower | 95% CI Upper | *p*-value | Weight |
| --- | --- | --- | --- | --- | --- |
| Camel Hump Oil |  |  |  |  |  |
| Alshaikhsaleh et al. 2025 | 2.85 | 1.92 | 3.78 | **<0.001** | 8.2% |
| Jassim et al. 2018 | 1.35 | 0.45 | 2.25 | **0.003** | 8.0% |
| Subtotal (I² = 78.2%) | **2.10** | **0.86** | **3.34** | **0.001** | **16.2%** |
| Camel Milk |  |  |  |  |  |
| Behrouz et al. 2024 | 1.45 | 0.68 | 2.22 | **<0.001** | 9.1% |
| Aljutaily et al. 2022 | 1.25 | 0.52 | 1.98 | **0.001** | 9.3% |
| El-Sawy et al. 2018 | 0.90 | 0.15 | 1.65 | **0.019** | 9.0% |
| Subtotal (I² = 45.1%) | **1.20** | **0.58** | **1.82** | **<0.001** | **27.4%** |
| Plant-Based Oils |  |  |  |  |  |
| Karvonen et al. 2002 | 0.85 | 0.35 | 1.35 | **0.001** | 12.5% |
| Musazadeh et al. 2021 | 1.05 | 0.60 | 1.50 | **<0.001** | 13.8% |
| Dobrzyńska et al. 2020 | 0.95 | 0.40 | 1.50 | **0.001** | 11.5% |
| Bellien et al. 2022 | 0.85 | 0.30 | 1.40 | **0.002** | 11.6% |
| Subtotal (I² = 52.3%) | **0.95** | **0.30** | **1.60** | **0.004** | **49.4%** |
| Overall Effect | **1.42** | **0.85** | **1.99** | **<0.001** | **100.0%** |

**Supplementary Table 4:** Forest Plot Data of MDA Activity.

| Study / Subgroup | SMD | 95% CI Lower | 95% CI Upper | p-value | Weight |
| --- | --- | --- | --- | --- | --- |
| Camel Hump Oil |  |  |  |  |  |
| Alshaikhsaleh et al. 2025 | -1.80 | -2.90 | -0.70 | **0.001** | 10.0% |
| Jassim et al. 2018 | -1.10 | -2.00 | -0.20 | **0.016** | 12.0% |
| Subtotal (I² = 75%) | **-1.45** | **-2.38** | **-0.52** | **0.002** | **22.0%** |
| Camel Milk |  |  |  |  |  |
| Behrouz et al. 2024 | -1.60 | -2.50 | -0.70 | **<0.001** | 11.0% |
| Aljutaily et al. 2022 | -1.30 | -2.10 | -0.50 | **0.001** | 12.5% |
| El-Sawy et al. 2018 | -0.90 | -1.65 | -0.15 | **0.019** | 14.5% |
| Subtotal (I² = 70%) | **-1.27** | **-1.85** | **-0.69** | **<0.001** | **38.0%** |
| Plant-Based Oils |  |  |  |  |  |
| Musazadeh et al. 2021 | -0.85 | -1.35 | -0.35 | **0.001** | 15.0% |
| Dobrzyńska et al. 2020 | -0.75 | -1.30 | -0.20 | **0.008** | 14.0% |
| Bellien et al. 2022 | -0.70 | -1.25 | -0.15 | **0.013** | 11.0% |
| Subtotal (I² = 60%) | **-0.77** | **-1.10** | **-0.44** | **<0.001** | **40.0%** |
| Overall Effect | **-1.28** | **-1.80** | **-0.76** | **<0.001** | **100.0%** |

**Supplementary Table 5:** Forest Plot Data of LDL-C.

| Study / Subgroup | SMD | 95% CI Lower | 95% CI Upper | p-value | Weight |
| --- | --- | --- | --- | --- | --- |
| Camel Hump Oil |  |  |  |  |  |
| Alshaikhsaleh et al. 2025 | -2.68 | -4.28 | -1.08 | **0.001** | 8.0% |
| Camel Milk |  |  |  |  |  |
| Behrouz et al. 2024 | -1.28 | -2.40 | -0.16 | **0.025** | 12.0% |
| Aljutaily et al. 2022 | -0.95 | -1.80 | -0.10 | **0.028** | 14.0% |
| Subtotal | **-1.12** | **-1.95** | **-0.29** | **0.008** | **26.0%** |
| Plant-Based Oils |  |  |  |  |  |
| Musazadeh et al. 2021 | -0.60 | -0.93 | -0.27 | **<0.001** | 20.0% |
| Dobrzyńska et al. 2020 | -0.40 | -0.90 | 0.10 | 0.120 | 18.0% |
| Bellien et al. 2022 | -0.30 | -0.85 | 0.25 | 0.284 | 18.0% |
| Subtotal | **-0.44** | **-0.74** | **-0.14** | **0.004** | **56.0%** |
| Overall Effect | **-0.92** | **-1.35** | **-0.49** | **<0.001** | **100.0%** |

**Supplementary Table 5:** Forest Plot Data of HDL-C.

| Study / Subgroup | SMD | 95% CI Lower | 95% CI Upper | p-value | Weight |
| --- | --- | --- | --- | --- | --- |
| Camel Hump Oil |  |  |  |  |  |
| Alshaikhsaleh et al. 2025 | 1.10 | 0.30 | 1.90 | **0.007** | 15.0% |
| Camel Milk |  |  |  |  |  |
| Behrouz et al. 2024 | 0.90 | 0.20 | 1.60 | **0.012** | 16.0% |
| Aljutaily et al. 2022 | 0.75 | 0.10 | 1.40 | **0.024** | 17.0% |
| Subtotal | **0.82** | **0.35** | **1.29** | **0.001** | **33.0%** |
| Plant-Based Oils |  |  |  |  |  |
| Musazadeh et al. 2021 | 0.70 | 0.25 | 1.15 | **0.002** | 19.0% |
| Dobrzyńska et al. 2020 | 0.65 | 0.20 | 1.10 | **0.005** | 18.0% |
| Bellien et al. 2022 | 0.60 | 0.15 | 1.05 | **0.009** | 15.0% |
| Subtotal | **0.65** | **0.40** | **0.90** | **<0.001** | **52.0%** |
| Overall Effect | **0.85** | **0.40** | **1.30** | **<0.001** | **100.0%** |
